# Prognostic impact of MALs and potential immunotherapy targets in uveal melanoma

**DOI:** 10.1002/pdi3.54

**Published:** 2024-05-22

**Authors:** Jing Yang, Zhou Fu, Qin Xiang

**Affiliations:** ^1^ Ophthalmology Department Children's Hospital of Chongqing Medical University Chongqing China; ^2^ National Clinical Research Center for Child Health and Disorders Ministry of Education Key Laboratory of Child Development and Disorders Chongqing Key Laboratory of Pediatrics Chongqing China

**Keywords:** immunotherapy target, MAL proteolipid family, prognosis biomarker, uveal melanoma

## Abstract

Uveal melanoma (UM) is the most common primary ocular malignancy in adults, and the 5‐year disease‐related mortality rate is 30%. MAL proteolipid family (MALs), including T‐cell differentiation protein (MAL), T‐cell differentiation protein 2 (MAL2), and T‐cell differentiation protein like (MALL), were involved in the progression and prognosis of many different cancers. However, the role of MALs in UM was not reported. UM samples were extracted from The Cancer Genome Atlas. R software (R3.6.3) was used to comprehensively analyze the roles of the MALs (signiﬁcance threshold: *p* < 0.05). MALs mRNA expression was changed in UM tissues. In terms of tumor stage, MAL2 was highly expressed in T4 (*p* = 0.021). The ROC curves indicated that MAL2 and MALL were prognostic biomarkers for 1‐ and 3‐year survival in UM patients, and MAL2 also could predict 5‐year survival for UM patients. Then, the univariable and multivariable analysis showed that MAL2 and MALL were independent prognostic biomarkers. Next, we assessed the immune microenvironment of MALs in UM. MAL had no correlation with B7‐H3, but MAL2 and MALL had a positive correlation with B7‐H3. Our results revealed that the MAL proteolipid family may be prognostic biomarkers for UM patients and that B7‐H3 may be a novel immunotherapy target for UM.

## Graphical abstract

Our results revealed that MAL, MAL2, and MALL may be prognostic biomarkers for UM patients and that B7‐H3 may be a novel immunotherapy target for UM.

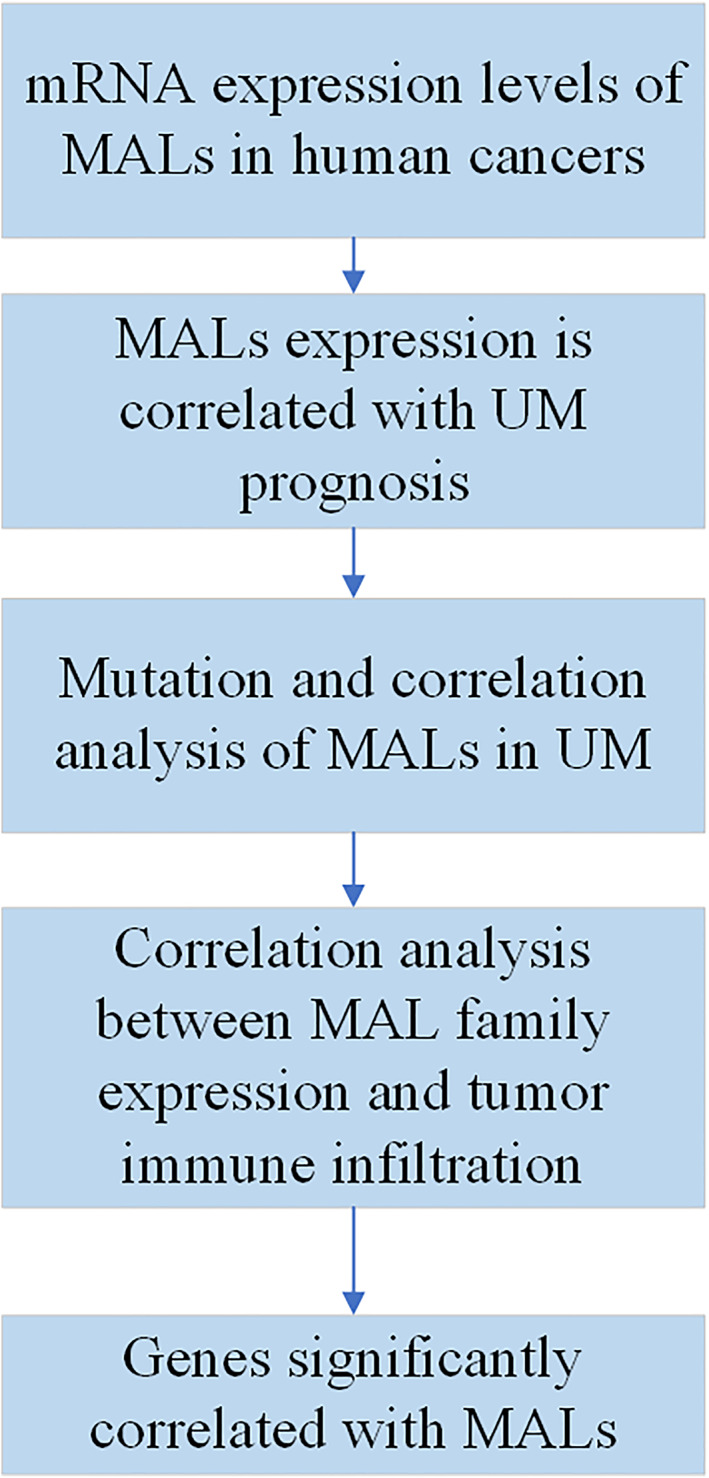

## 1 INTRODUCTION

Uveal melanoma (UM) is the most common primary ocular malignancy in adults^1^, ^2^ and is the second most common melanoma subtype after cutaneous melanoma.^3^ It originates from melanocytes in the uvea, which includes the pigmented tissues of the choroidal plexus, ciliary body, or iris of the eye.^4^ UM presents with various symptoms, such as blurred or distorted vision, visual field loss or photopsia, but approximately 30% of patients with UM are asymptomatic. Despite treatment of the primary tumor, almost 50% of UM patients will develop metastatic disease, which is usually fatal after 1 year.^5^ Although UM is common in adults, children can also develop it.^6^ To date, only a few studies have revealed high‐risk and low‐risk loci. Therefore, it is urgent to identify the molecular markers that mediate the pathogenesis to improve treatment selection and prognosis in UM patients.

The family of MAL proteolipids (MALs) include T‐cell differentiation protein (MAL), T‐cell differentiation protein 2 (MAL2), and T‐cell differentiation protein like (MALL). MALs are localized to the endoplasmic reticulum of T cells and are candidate linker proteins in T‐cell signal transduction. Previous studies have reported that downregulation of MAL or MAL2 is associated with a variety of human epithelial malignancies.^7^, ^8^, ^9^ Studies have confirmed that the analysis of MAL gene methylation, the measurement of the expression of MAL mRNA levels and the immunohistochemical detection of the MAL protein are useful diagnostic tools for defining cancer cell subpopulations that are susceptible to becoming malignant and to metastasizing or for use as prognostic biomarkers.^10^ These studies suggested MALs could be used as a tumor monitoring indicator. However, no study has focused on MALs and UM. Therefore, we further explored the correlation between MALs family and UM.

In this study, we aimed to demonstrate the relationship between MALs and UM based on RNA‐sequencing data from The Cancer Genome Atlas (TCGA). Our work first revealed that increased expression of MALs is linked to poor overall survival (OS) among UM patients. Moreover, we used enrichment analysis and immune infiltration correlation analysis to define the biological significance of MALs. Overall, this work revealed the role of MALs in UM development, which will help to elucidate the mechanisms underlying MALs.

## 2 MATERIALS AND METHODS

### 2.1 RNA‐seq data source

Data on a total of 80 UM cases with gene expression data (HTSeq‐FPKM) were collected from the TCGA. Samples were divided into a high expression group and a low expression group according to the median MAL expression level, MAL2 expression level, and MALL expression level. This study met the publication guidelines stated by TCGA (https://www.cancer.gov/) and GTEx (http://gepia.cancer‐pku.cn/). All data used were acquired from TCGA and GTEx, and ethical approval and informed consent of the patients were not required.^11^

### 2.2 Correlation between clinical characteristics and MALs

The ggplot2 package^12^ was used to visualize the relationship between pathologic T stage and MALs. The survminer package and survival package^13^ were used to perform survival analysis and Cox univariate and multifactorial regression analyses, and the survminer package was utilized to visualize the survival curve of OS with the log‐rank test and Cox analysis. The TimeROC package^13^ was utilized to generate a time‐dependent operated characteristic curve (ROC) of OS.

### 2.3 Evaluation of immune cell infiltration in tumor tissue

Tumor immune infiltration was identified with reference to previous studies.^14^ ssGSEA algorithm from the R language GSVA package^15^ was used to assess the immune stromal component of the tumor microenvironment for each sample. The greater the ratio of the relevant component, the higher the correlation between immune cells and UM.

### 2.4 Gene correlation analysis

Correlations between MALs and other genes were assessed using Spearman's correlation coefficient. Statistical analysis was performed and graphs were generated with the stat package of R software by R3.6.3. *p* < 0.05 was considered significant.

### 2.5 Enrichment analysis

Differentially expressed genes were acquired between the high and low MAL family expression groups. Then, GO analysis and KEGG enrichment analysis were performed to determine the biological processes, molecular function, cellular composition, and signaling pathways related to the differentially expressed genes.

### 2.6 Statistical analyses

Statistical analysis was performed using the stat package of R software by R3.6.3. The Mann‒Whitney *U* test was used to analyze gene expression levels between cancer and normal tissue. The Kruskal‒Wallis test was used to compare gene expression levels in the two groups of UM, and correlations were analyzed using Spearman's correlation coefficient. The log‐rank test was used to assess the K–M survival curve. *p* < 0.05 was considered to indicate statistical significance.

## 3 RESULTS

### 3.1 mRNA expression levels of MALs in human cancers

**FIGURE 1:**
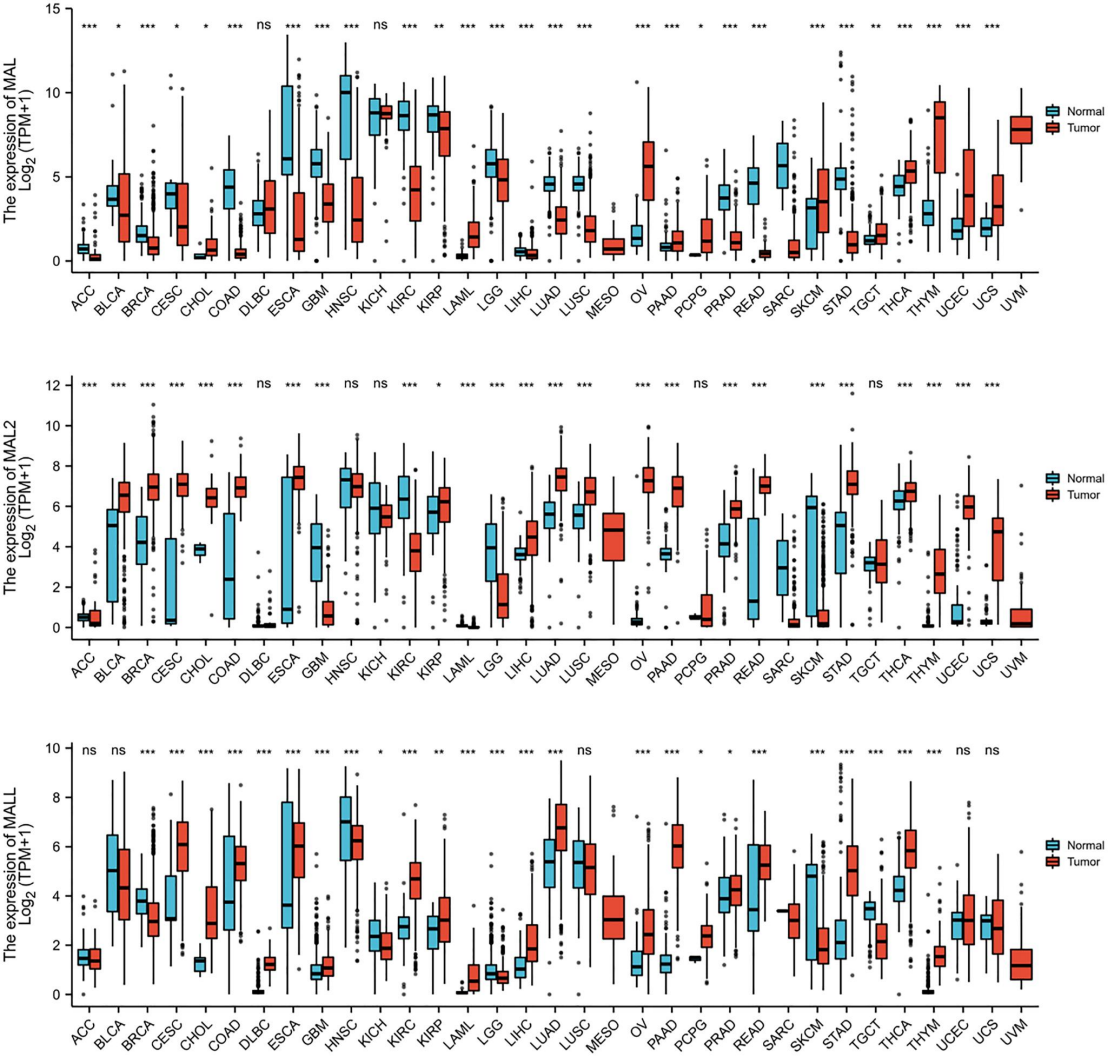
mRNA expression levels of MAL, MAL2, and MALL in human cancers. ACC, adrenocortical carcinoma; BLCA, bladder urothelial carcinoma; BRCA, breast invasive carcinoma; CESC, cervical squamous cell carcinoma and endocervical adenocarcinoma; CHOL, cholangio carcinoma; COAD, colon adenocarcinoma; DLBC, lymphoid neoplasm diffuse large B‐cell lymphoma; ESCA, esophageal carcinoma; GBM, glioblastoma multiforme; HNSC, head and neck squamous cell carcinoma; KICH, kidney chromophobe; KIRC, kidney renal clear cell carcinoma; KIRP, kidney renal papillary cell carcinoma; LAML, acute myeloid leukemia; LGG, brain lower grade glioma; LIHC, liver hepatocellular carcinoma; LUAD, lung adenocarcinoma; LUSC, lung squamous cell carcinoma; MESO, mesothelioma; OV, ovarian serous cystadenocarcinoma; PAAD, pancreatic adenocarcinoma; PCPG, pheochromocytoma and paraganglioma; PRAD, prostate adenocarcinoma; READ, rectum adenocarcinoma; SARC, sarcoma; SKCM, skin cutaneous melanoma; STAD, stomach adenocarcinoma; TGCT, testicular germ cell tumor; THCA, thyroid carcinoma; THYM, thymoma; UCEC, uterine corpus endometrial carcinoma; UCS, uterine carcinosarcoma; UVM, uveal melanoma. *p < 0.05; **p < 0.01; ***p < 0.001.

The mRNA expression levels of MALs in cancer and normal tissues were determined using UCSC XENA (https://xenabrowser.net/datapages/) (Figure 1). The mRNA expression levels of MAL were significantly different between cancer tissue and normal tissue; they were downregulated in 18 cancers and upregulated in 12 cancers. MAL2 was found to be significantly downregulated in 11 analyses and upregulated in 19 analyses. The results showed that the mRNA expression levels of MALL were significantly downregulated in 6 analyses and upregulated in 19 analyses. However, there were no control samples of normal tissues in the TCGA‐UM dataset. The expression level of MALs also changed in UM tissues. The expression of MAL was high in UM, but the expression of MAL2 and MALL was low in UM.

### 3.2 Prognostic implications of MAL family expression in UM

**FIGURE 2:**
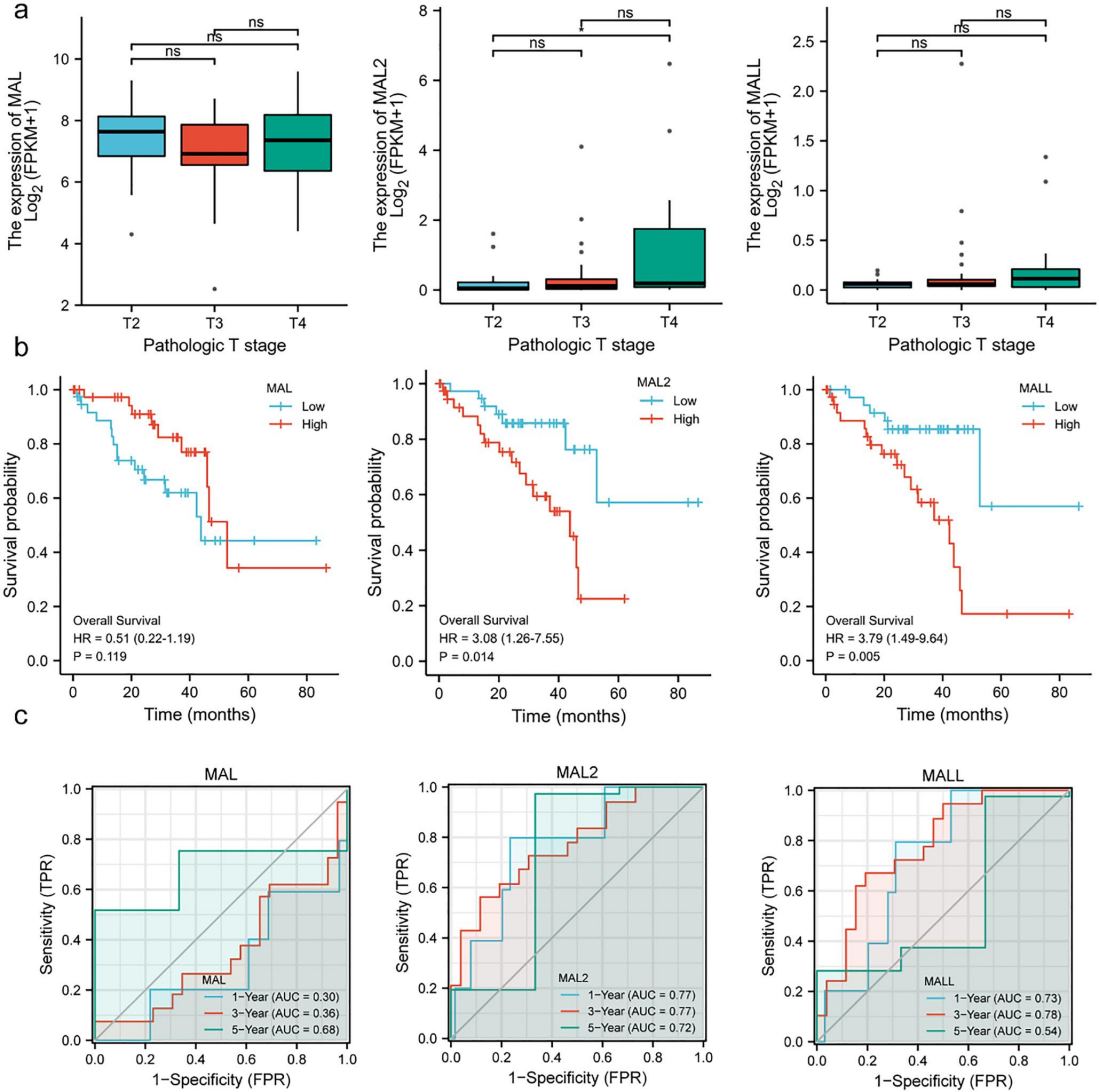
The MAL family expression is correlated with UM prognosis. (A) The correlation between pathologic T stage and MALs expression. (B) Effect of MALs on OS in UM. (C) Time‐dependent ROC curve of the expression of MALs on OS in UM. *p < 0.05. OS, overall survival; UM, uveal melanoma.

The relationship between tumor stage and MAL family expression was examined using the TCGA database (Figure 2A). A total of 80 UM patients had definite pathologic tumor stage data; there were 14 patients with T2, 32 with T3, and 34 with T4 stage disease. The results revealed that MAL2 upregulated in T4 samples (*p* = 0.021) compared to T2 samples. Although the expression level of MAL2 in T3 was not statistically significant compared to T4 samples, we found the tendency that the median expression level of MAL2 in T3 was slightly higher than that in T2 and slightly lower than that in T4. No significant correlations were observed between MAL/MALL expression and tumor stage.

We also evaluated the effect of MAL and MAL2 on the prognosis of UM using Kaplan‒Meier Plotter (Figure 2B). These data showed that patients in the MAL2 low‐expression group had better OS than those in the MAL2 high‐expression group (*p* = 0.014), and patients in the MALL low‐expression group had better OS than those in the MALL high‐expression group. However, no significant correlations were observed between MAL and OS.

Then, the timeROC package was used to analyze the time‐dependent ROC curve indicating the ability of the expression of MALs to predict OS in UM patients (Figure 2C). For MAL, the area under the curve values for 1‐, 3‐, and 5‐year survival were 0.302, 0.355, and 0.675, respectively, with 95% confidence intervals of 0.041–0.563, 0.177–0.534, and 0.450–0.901, respectively. For MAL2, the area under the curve values for 1‐, 3‐, and 5‐year survival were 0.769, 0.773, and 0.722, respectively, and the 95% confidence intervals were 0.575–0.963, 0.631–0.915, and 0.293–1.151, respectively. For MALL, the area under the curve values for 1‐, 3‐, and 5‐year survival were 0.726, 0.780, and 0.544, respectively, and the 95% confidence intervals were 0.559–0.893, 0.645–0.916, and 0.163–0.927, respectively.

Further univariable analysis showed that pathologic M stage (M0 vs. M1), pathologic stage (stage II vs. stage IV), histological type (epithelioid cell vs. spindle cell), tumor shape (diffuse vs. dome, diffuse vs. mushroom), MAL2 level (low vs. high), and MALL level (low vs. high) were prognostic factors predicting OS in UM patients (Supplementary Table S1). Multivariable analysis showed that older age, epithelioid histological type, and spindle cell histological type were significantly associated with the risk of death. These data suggested that MALs could be used as prognostic biomarkers for UM patients.

### 3.3 Mutation and correlation analysis of MALs

**FIGURE 3:**
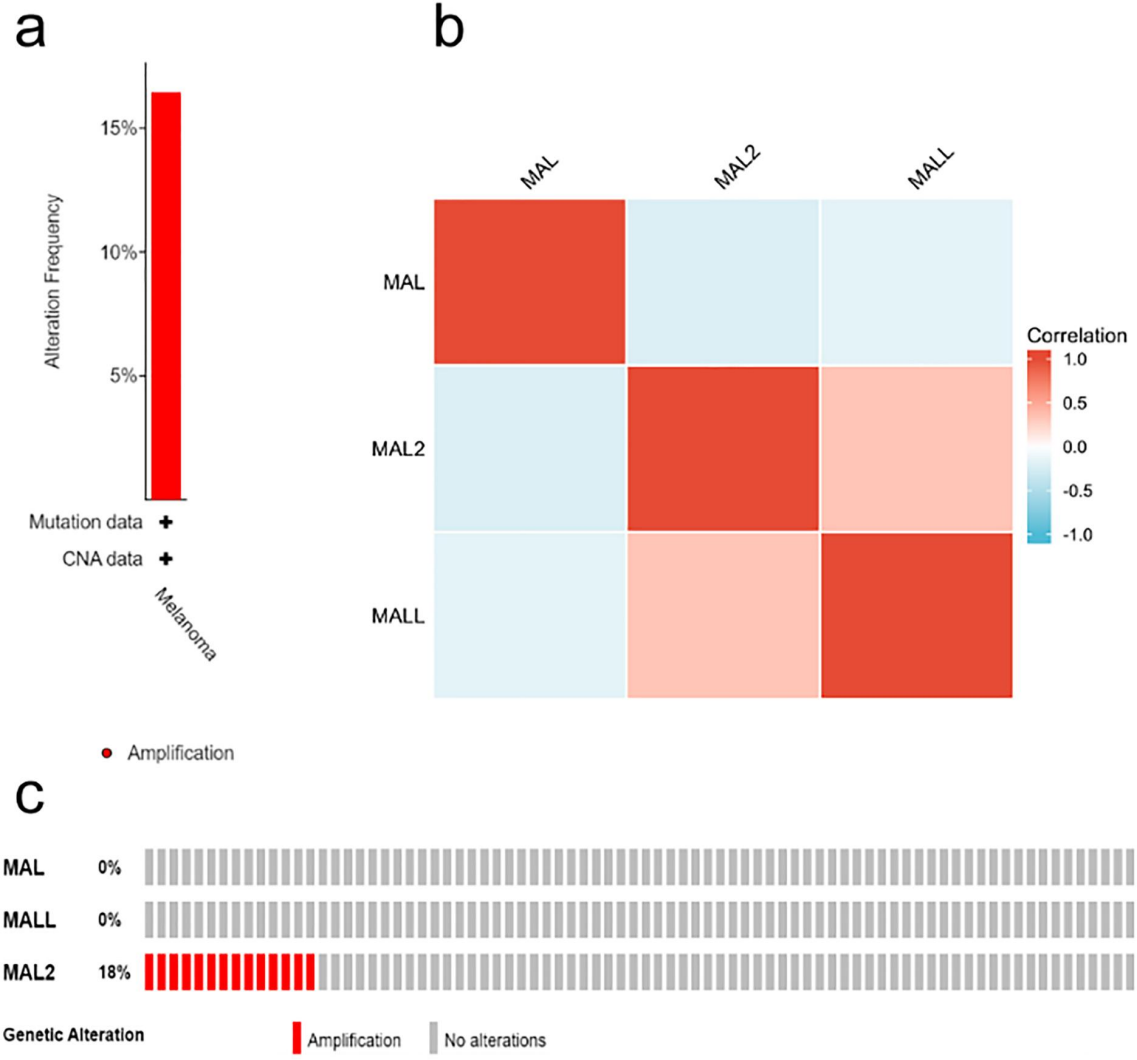
Mutation and correlation analysis of MALs in UM. (A) Mutation frequency of MALs. (B) Correlation between every two MALs. (C) Mutation details of every MAL family member in each individual sample. UM, uveal melanoma.

The cBioPortal database was utilized to investigate the mutational landscape of MAL, MAL2, and MALL. Eighteen percent (14/80) of patients had genetic alterations, and amplification was the most frequent mutation type (Figure 3A). MAL2 had only amplification mutations, and MAL and MALL had no alterations (Figure 3C). Next, we examined the correlations among the MAL members using Pearson correlation analysis (Figure 3B). The results showed that MAL2 and MALL had a significant positive correlation, and a negative correlation was observed between MAL and MAL2.

### 3.4 The correlation between MAL family expression and the immune microenvironment in UM

**FIGURE 4:**
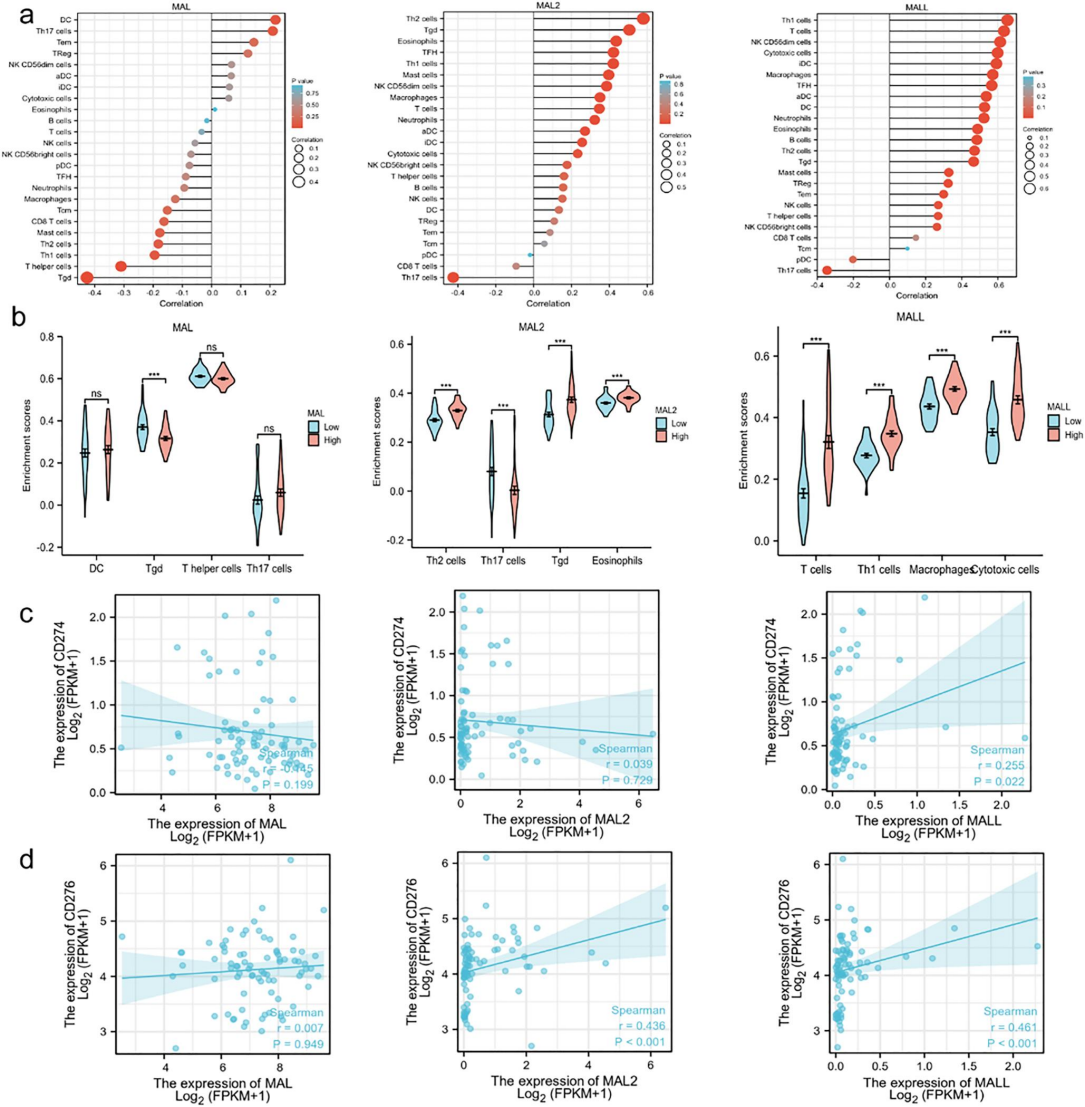
(A) Correlation analysis between the MAL family expression and tumor immune infiltration. (B) The distribution of tumor immune infiltrates in groups with high and low expression of MALs. (C) Correlation analysis of PD‐L1 and MALs. (D) Correlation analysis of B7‐H3 and MALs. aDC, activated DC; B cell, CD8 T cells; iDC, immature DC; pDC, Plasmacytoid DC; Tcm, T central memory; Tem, T effector memory; Tfh, T follicular helper; Tgd, T gamma delta. *p < 0.05; **p < 0.01; ***p < 0.001.

The ssGSEA algorithm from the R language GSVA package ssGSEA package was used to analyze the correlation between MAL family expression levels and tumor immune infiltration, and the results were calculated by the Spearman method. This result suggested that MAL expression was positively correlated with the infiltration of DCs and Th17 cells and negatively correlated with that of Tgd and T helper cells (Figure 4A). MAL2 expression was positively correlated with the infiltration of Th2, Tgd, and eosinophils and negatively correlated with the infiltration of Th17 cells. MALL expression was positively correlated with the infiltration of Th1 cells, T cells, and cytotoxic cells and negatively correlated with the infiltration of Th17 cells. The GSVA package was used to investigate the distribution of tumor immune infiltration in samples with high and low expression of MALs (Figure 4B). The results showed that the infiltration of Tgd cells was higher in samples with low MAL expression than in those with high MAL expression, but the infiltration of Tgd cells in samples with low MAL2 expression was lower than in samples with high MAL2 expression. T cell infiltration was lower in the low MALL group than in the high MALL group. These results suggest that MAL, MAL2, and MALL expression levels are significantly associated with the characteristics of the UM immune microenvironment, and the correlation between MAL expression and immune microenvironment factors was weaker than that between MAL2 and MALL expression and immune microenvironment factors.

Next, the ggplot2 package was used to visualize the correlation between MALs and some novel immunotherapy targets, such as PD‐L1 (CD274) and B7‐H3 (CD276). We found that MAL and MAL2 had no correlation with PD‐L1, but MALL had a positive correlation with PD‐L1 (Figure 4C). MAL had no correlation with B7‐H3, but MAL2 and MALL had a positive correlation with B7‐H3 (Figure 4D). These results suggest that B7‐H3 may be a novel immunotherapy target for UM.

### 3.5 Genes significantly correlated with MALs

**FIGURE 5:**
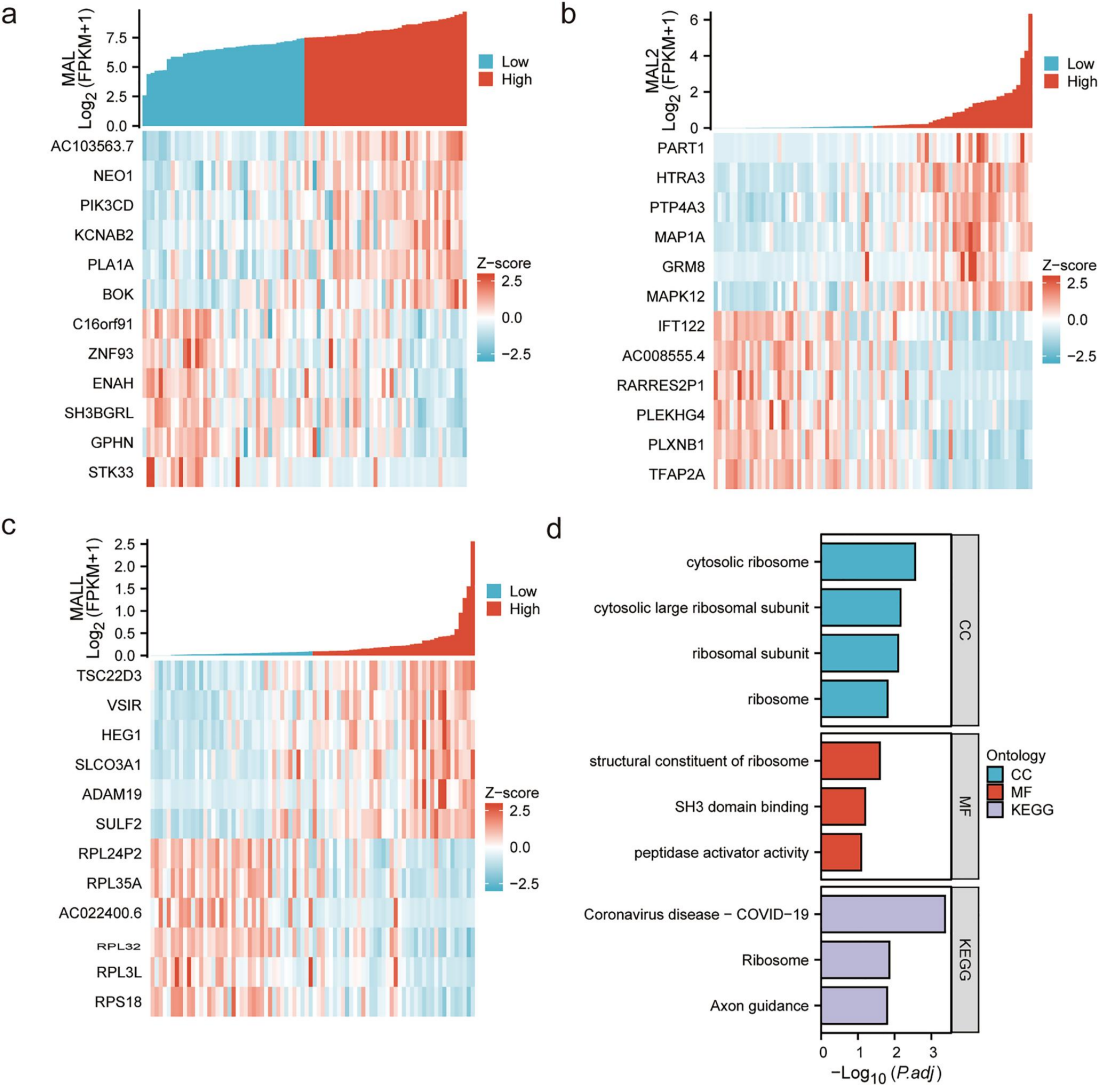
Genes significantly correlated with MALs. (A) Heatmap of the top 12 genes correlated with MAL. (B) Heatmap of the top 12 genes correlated with MAL2. (C) Heatmap of the top 12 genes correlated with MALL. (D) GO and KEGG enrichment analyses of genes correlated with MALs.

Spearman's test was used to analyze the genes significantly correlated genes with MALs. The top 6 correlated genes are shown in the heatmap plot (Figure 5A–C). We found that the most positively correlated genes with MAL were STK33, GPHN, and SH3BGRL, while the negatively correlated genes were AC103563.7, NEO1, and PIK3CD. The most negatively correlated genes with MAL2 were TFAP2A, PLXNB1, and PLEKHG4, while the positively correlated genes were PART1, HTRA3, and PTP4A3. The most positively correlated genes with MALL were TSC22D3, VSIR, and HEG1, while the negatively correlated genes were RPL32, RPL3L, and RPS18.

Next, GO and KEGG enrichment analyses were utilized to analyze MALs and the above correlated genes (39 genes in total) (Figure 5D). The GO analysis results showed that cytosolic ribosome, cytosolic large robosomal subunit, and structural constituent of ribosome were the main associated pathways. Axon guidance and ribosomes were enriched according to KEGG enrichment analysis.

## 4 DISCUSSION

The rate of UM metastasis is approximately 25% at 5 years, and the 5‐year survival rate for primary UM patients ranges from only 17%–53%.^3^, ^16^, ^17^ Tracking treatment patterns and survival in patients with UM will become increasingly important as new treatments are developed and our ability to guide treatment decision making based on metastatic risk profile improves.^18^ Therefore, there is still a need to find more prognostic biomarkers for UM patients.^19^, ^20^ MALs are hot spot in tumor research at present. Dersh and Yewdell^21^ found that the MAL2 expression was associated with a poor prognosis for breast cancer, and its downregulation enhanced CD8+ T‐cell recognition of breast cancer in various experimental models. Choi et al.^22^ found that MAL was a novel DNA methylation marker in human gastric cancer. Therefore, we hypothesize that the MAL family may play a vital role in tumor patients.

We first evaluated the mRNA expression levels of MALs in human cancers and found that the expression of MALs differs from tumor to tumor. Studies have shown that MALs were involved in the progression and prognosis of many cancers, and we found the expression level of MALs also changed in UM. However, the role of MALs family in UM was not reported. Therefore, we further explored the correlation between the MALs family and UM. Then, we utilized 80 UM samples obtained from the TCGA database. By studying the ROC curve analysis and univariable analysis, we found that MALs could be used as prognostic biomarkers for UM patients. MAL2 and MALL were prognostic biomarkers for predicting the probability of survival at one and 3 years for UM patients, and MAL2 was also a prognostic biomarker for predicting the probability of survival at 5 years.

By promoting antitumor immune responses, immunotherapy has revolutionized the treatment of cancer over the past decade by producing significant and durable responses in patients with many different types of tumors.^23^ Cancer immunotherapy based on immune evasion mechanisms (e.g., anti‐PD therapy) achieved higher objective response rates in patients with immune‐related adverse events than in those with an immunosuppressive phenotype.^24^ B7‐H3 (CD276) is highly expressed in different types of human cancers, and there are a number of clinical trials on anti‐CD276 antibodies for hematologic and solid tumor malignancies.^25^ However, no study has reported the therapeutic effect of CD276 in UM. To screen the target of immunotherapy, we further analyzed the immune microenvironment and its correlation with MALs in UM. The results suggested that MAL had no correlation with B7‐H3, but MAL2 and MALL had a positive correlation with B7‐H3. These results suggested that B7‐H3 may be a novel immunotherapy target for UM.

There are some limitations to our study. First, we only analyzed the data from the TCGA database with a limited sample size; therefore, other related data should be further collected to screen more prognostic biomarkers for UM patients. Second, due to the rarity of UM, it was difficult for us to obtain tissue samples, so we only conducted high‐throughput mRNA‐seq profiling data analysis. In addition, to deeply understand the mechanism, it is necessary to further functionally verify the importance of MALs in the prognosis of UM. Last, we have not shown a correlation between B7‐H3 and MALs, so more work should be done to research the immunotherapy effect of B7‐H3 for UM.

In summary, our results revealed that MAL, MAL2, and MALL are potential prognostic biomarkers for UM patients and that B7‐H3 (CD276) could be a potential immunotherapy target.

## AUTHOR CONTRIBUTIONS

Qin Xiang was responsible for the overall conception and design of the study and for revising the manuscript. Jing Yang performed the data analysis, collected the data, and wrote the manuscript. Zhou Fu prepared Figures 1, 2, 3, 4, 5. All authors reviewed, edited and approved the manuscript.

## CONFLICT OF INTEREST STATEMENT

We declare no conflict of interest.

## ETHICS STATEMENT

Not applicable.

## Supporting information

Table S1

## Data Availability

The data that support the findings of this study are available in TCGA database at https://portal.gdc.cancer.gov/. These data were derived from the following resources available in the public domain: TCGA database, https://portal.gdc.cancer.gov/.
