# Supplementary material for: Prognostic impact of MALs and potential immunotherapy targets in uveal melanoma
Source: Pediatr Discov. 2024 May 22;2(3):e54. doi: 10.1002/pdi3.54 (PMC12118173; doi:10.1002/pdi3.54)
Supplement: Supplementary file 1 — Table S1 [file PDI3-2-e54-s001.docx]

**Supplementary Table 1.** Univariable and multivariable analysis of OS in patients with UM.

| Variable |  | Number | Univariable analysis | | | Multivariable analysis | |
| --- | --- | --- | --- | --- | --- | --- | --- |
|  |  |  | HR (95% CI) | *P* value | | HR (95% CI) | *P* value |
| Age |  | 80 |  | |  |  |  |
|  | ≤60 | 40 | Reference | |  | Reference |  |
|  | >60 | 40 | 2.123(0.914-4.933) | | 0.080 | 9.818(1.234-78.087) | 0.031 |
| Sex |  | 80 |  | |  |  |  |
|  | Female | 35 | Reference | |  |  |  |
|  | Male | 45 | 1.542(0.651-3.652) | | 0.325 |  |  |
| Pathologic T stage |  | 80 |  | |  |  |  |
|  | T2 | 14 | Reference | |  |  |  |
|  | T3 | 32 | 3.138(0.401-24.558) | | 0.276 |  |  |
|  | T4 | 34 | 4.572(0.590-35.428) | | 0.146 |  |  |
| Pathologic N stage |  | 79 |  | |  |  |  |
|  | N0 | 52 | Reference | |  |  |  |
|  | NX | 27 | 0.890(0.360-2.198) | | 0.800 |  |  |
| Pathologic M Stage |  | 78 |  | |  |  |  |
|  | M0 | 51 | Reference | |  | Reference |  |
|  | M1 | 4 | 54.348(5.517-535.347) | | <0.001 | 30688918719.707(0.000-lnf) | 0.998 |
|  | MX | 23 | 0.521(0.171-1.587) | | 0.252 | 0.102(0.007-1.405) | 0.088 |
| Pathologic stage |  | 79 |  | |  |  |  |
|  | Stage II | 39 | Reference | |  | Reference |  |
|  | Stage III | 36 | 1.180(0.474-2.940) | | 0.722 | 0.463(0.074-2.906) | 0.411 |
|  | Stage IV | 4 | 69.945(6.778-721.852) | | <0.001 |  |  |
| Histological type |  | 80 |  | |  |  |  |
|  | Epithelioid cell | 13 | Reference | |  | Reference |  |
|  | Spindle cell | 30 | 0.107(0.025-0.452) | | 0.002 | 0.029(0.002-0.498) | 0.015 |
|  | Mix | 37 | 0.438(0.157-1.218) | | 0.113 | 0.098(0.018-0.522) | 0.007 |
| Tumor shape |  | 51 |  | |  |  |  |
|  | Diffuse | 2 | Reference | |  | Reference |  |
|  | Dome | 36 | 0.017(0.002-0.197) | | 0.001 | 0.000(0.000-lnf) | 0.998 |
|  | Mushroom | 13 | 0.010(0.001-0.138) | | <0.001 | 0.000(0.000-lnf) | 0.998 |
| MAL |  | 80 |  | |  |  |  |
|  | Low | 40 | Reference | |  | Reference |  |
|  | High | 40 | 0.513(0.222-1.187) | | 0.119 |  |  |
| MAL2 |  | 80 |  | |  |  |  |
|  | Low | 40 | Reference | |  | Reference |  |
|  | High | 40 | 3.080(1.256-7.551) | | 0.014 | 7.208(0.970-53.570) | 0.261 |
| MALL |  | 80 |  | |  |  |  |
|  | Low | 40 | Reference | |  | Reference |  |
|  | High | 40 | 3.788(1.489-9.635) | | 0.005 | 4.253(0.341-53.074) | 0.261 |
